# Angiogenic Biomarkers and Neonatal Outcomes in Suspected Preeclampsia: Retrospective Cohort Study

**DOI:** 10.1111/1471-0528.70104

**Published:** 2025-12-08

**Authors:** Genevieve P. G. Fung, Hugh S. Lam, Kathy Y. Y. Chan, Isabella Y. M. Wah, Caitlyn S. L. Lau, Long Nguyen‐Hoang, Daljit S. Sahota, Liona C. Poon

**Affiliations:** ^1^ Department of Paediatrics The Chinese University of Hong Kong Hong Kong SAR China; ^2^ Department of Obstetrics and Gynaecology The Chinese University of Hong Kong Hong Kong SAR China; ^3^ Shenzhen Research Institute The Chinese University of Hong Kong Hong Kong SAR China

**Keywords:** adverse outcome, fms‐like tyrosine kinase 1, neonate, placental growth factor, PlGF, preeclampsia, ratio, sFlt‐1

## Abstract

Placental growth factor (PlGF) and soluble fms‐like tyrosine kinase 1 (sFlt1) are two biomarkers associated with placental function and the sFlt‐1/PlGF ratio is frequently used for risk assessment and prediction of preeclampsia in pregnancy.

**Objective:**

To assess the association between elevated sFlt‐1/PlGF ratio and adverse neonatal outcomes in pregnancies with suspected preeclampsia.

**Design:**

Retrospective cohort study.

**Setting:**

A tertiary centre in Hong Kong.

**Population:**

162 singleton neonates delivered between January 2020 and May 2023 from pregnancies complicated by suspected preeclampsia and sFlt‐1/PlGF taken within 4 weeks before delivery.

**Methods:**

Pregnant women with suspected preeclampsia were recruited with analysis of serum Flt‐1 and PlGF. Neonatal outcomes were compared between neonates from pregnancies with sFlt‐1/PlGF ratio ≥ 85 (high‐risk group) and < 85 (low‐risk group).

**Main Outcome Measures:**

Neonatal outcomes including growth, respiratory, gastrointestinal, and metabolic complications.

**Results:**

Logistic regression analysis showed that after adjustment for gestational age, preeclampsia and newborn sex, sFlt‐1/PlGF ratio ≥ 85 was significantly associated with small‐for‐gestation (SGA; aOR = 2.52, 95% C.I. = 1.12–5.60), feeding intolerance (aOR = 3.03, 95% C.I. = 1.01–9.09), need for parenteral nutrition (PN; aOR = 11.19, 95% C.I. = 2.23–56.29), prolonged PN (aOR = 6.92, 95% C.I. = 1.43–33.39), hospitalisation over 7 and 30 days (aOR = 3.62, 95% C.I. = 1.20–10.87 and aOR = 7.04, 95% C.I. = 1.08–45.80 respectively). Linear regression showed a significant association between sFlt‐1/PlGF ratio and duration of respiratory support, PN and duration of hospital stay.

**Conclusions:**

An elevated maternal sFlt‐1/PlGF ratio within 4 weeks of delivery is significantly associated with adverse neonatal outcomes including SGA, gastrointestinal complications, prolonged parenteral nutrition and prolonged hospitalisation.

## 1 Introduction

Preeclampsia affects 2%–5% of pregnancies worldwide and is one of the most common causes of maternal and fetal morbidity and mortality [1]. Approximately 90% of cases occur at or after 34 weeks' gestation, while 10% occur before 34 weeks [1, 2]. Preeclampsia is associated with multi‐organ dysfunction in the mother, with adverse maternal and fetal sequelae [3, 4, 5]. In utero exposure to preeclampsia is associated with an increased risk of stillbirth and preterm delivery. Early‐onset preeclampsia before 34 weeks' gestation is more commonly linked to fetal growth restriction and small‐for‐gestational‐age (SGA) neonates [4].

Identification of early predictors of preeclampsia is important for timely management to optimise maternal and neonatal outcomes [3, 6, 7, 8]. Two important angiogenic biomarkers produced by the placenta: soluble fms‐like tyrosine kinase 1 (sFlt‐1) and placental growth factor (PlGF), play significant roles in the development of preeclampsia [9, 10]. In pregnant women, an elevated sFlt‐1/PlGF ratio is associated with systemic vasoconstriction, renal glomerular damage, cerebral oedema, and endothelial dysfunction manifesting clinically as hypertension, proteinuria and headache [1, 11, 12].

An optimal balance of sFlt‐1 and PlGF is important for healthy placental vascular and endothelial development [13, 14]. The PROGNOSIS and PROGNOSIS ASIA studies [15, 16] validated a sFlt‐1/PlGF ratio of ≤ 38 to rule out preeclampsia, whilst ratios ≥ 78 and ≥ 85 were associated with imminent disease onset [17, 18, 19]. Recent studies have reinforced the clinical utility of angiogenic markers. Wright et al. [20] demonstrated that racial and ethnic differences may influence biomarker interpretation, thus emphasising the importance of population‐specific thresholds for sFlt‐1 and PlGF measurements. Magee et al. [21] showed that PlGF testing between 19 and 23 weeks can guide subsequent care and improve pregnancy outcomes. These findings support the integration of angiogenic markers into screening and risk stratification protocols.

An elevated sFlt‐1/PlGF ratio has also been observed in cases of isolated fetal growth restriction in the absence of overt preeclampsia. Witwicki et al. also demonstrated that elevated sFlt‐1/PlGF ratios in normotensive pregnancies were associated with increased risk of neonatal intensive care unit (NICU) admission, respiratory complications, and necrotising enterocolitis in SGA infants independent of maternal preeclampsia [22]. However, there are no large‐scale, comprehensive studies exploring the association between angiogenic markers, preeclampsia and various adverse outcomes in neonates.

The primary objective of this study is to assess the association between elevated sFlt‐1/PlGF ratios (≥ 85), measured within 4 weeks of delivery, and neonatal outcomes in pregnancies complicated by suspected preeclampsia. The secondary objective is to examine the relationship between sFlt‐1/PlGF ratios and specific adverse neonatal outcomes, including small‐for‐gestation, respiratory, gastrointestinal and metabolic complications, and prolonged hospitalisation.

## 2 Methods

This is a secondary analysis of a cohort study conducted between January 2020 and May 2023 at the Prince of Wales Hospital, Hong Kong, a tertiary referral center with Obstetrics and NICU support. Our hospital provides maternity services to a population of approximately 1.7 million with an annual delivery rate of 4000–5000 per year. The study protocol was approved by the Joint Chinese University of Hong Kong—New Territories East Cluster Clinical Ethics Committee (CREC Ref No: 2024.054) and was conducted according to the Declaration of Helsinki.

### 2.1 Subjects

#### 2.1.1 Mother‐Baby Dyads

East Asian women aged 18 years or older having a singleton pregnancy were recruited by the obstetrics team between 20 + 0 and 36 + 6 weeks of gestation to participate in a study on the prediction of preeclampsia if they had one or more of the following clinical features of suspected preeclampsia: (1) new onset or worsening of pre‐existing hypertension, (2) new onset proteinuria or worsening of pre‐existing proteinuria, (3) preeclampsia‐related maternal symptoms (including epigastric pain, excessive edema/severe swelling over face, hands and feet, sudden weight gain of > 1 kg/week in the third trimester, headache, visual disturbance and abnormal blood parameters including low platelet and elevated liver transaminase) or (4) fetal manifestations including fetal growth restriction. Pregnancies complicated with fetal chromosomal abnormalities, major structural abnormalities and stillbirth were excluded.

Following delivery, neonates were included if maternal sFlt‐1 and PlGF levels were measured within 4 weeks prior to birth. Neonates were excluded if they were (1) delivered at other hospitals, (2) sFlt‐1 or PlGF results were unavailable, (3) biomarker testing occurred more than 4 weeks before delivery and (4) neonatal clinical data were missing. Written informed consent was obtained from all women participating in the study at the time of recruitment.

New‐onset hypertension was defined as a confirmed single episode of systolic blood pressure (SBP) ≥ 140 mmHg and/or diastolic blood pressure (DBP) ≥ 90 mmHg. Worsening of pre‐existing hypertension was defined as a single measurement of SBP ≥ 160 mmHg and/or DBP ≥ 110 mmHg. New onset of proteinuria was defined as ≥ 1 + protein on urine dipstick test with urinary tract infection excluded. Worsening of pre‐existing proteinuria was defined as ≥ 2 + protein on urine dipstick test or ≥ 0.3 g protein over 24 h or spot urine protein/creatinine ratio ≥ 30 mg/mmol [23, 24]. All recruited participants underwent a structured assessment in accordance with our internally agreed and published departmental protocol for the management of suspected preeclampsia. Preeclampsia was diagnosed according to the 2018 International Society for the Study of Hypertension in Pregnancy (ISSHP) classification [24]. Women with confirmed diagnosis of preeclampsia at presentation were excluded from the study [25]. Serum samples for angiogenic markers (sFlt‐1 and PlGF) were collected at the time of hospital admission. The subsequent management of the women followed the established departmental protocol. Neonates were managed by the neonatal team after delivery according to our neonatal unit protocols.

### 2.2 Biomarkers sFlt‐1/PlGF Threshold Levels

Serum PlGF and sFlt‐1 concentrations were analysed using the fully automated Elecsys immunoassay platform (Roche Diagnostics, Indianapolis, USA). Results were reported in picograms per millilitre (pg/mL). The lower limit of detection (LLD) for PlGF was 3 pg/mL, and for sFlt‐1 was 10 pg/mL, as per manufacturer specifications. A sFlt/PlGF ratio of ≤ 38 has been validated to rule out preeclampsia (12), whilst a threshold value of ≥ 85 has been validated for the diagnosis of preeclampsia [6, 7, 8, 9, 10, 11, 12, 13, 14, 15, 16, 17, 18, 19, 20, 21, 22, 23, 24, 25, 26, 27, 28]. For this study, an elevated sFlt‐1/PlGF ratio was defined as sFlt‐1/PlGF ratio ≥ 85.

### 2.3 Neonatal Outcomes Assessment

All singleton neonates delivered from pregnant women with suspected preeclampsia with biomarkers sFlt‐1 and PlGF measured within 4 weeks of delivery were included in this study. Recruited neonates were categorised into (1) High‐risk group: neonates from pregnancies with an elevated sFlt‐1/PlGF ratio ≥ 85 and (2) Low‐risk group: neonates from pregnancies with a normal sFlt‐1/PlGF ratio < 85.

Neonatal data was retrieved from the Hospital Authority's “Clinical Management System”, which contains diagnosis and procedure coding, discharge summary, medications, laboratory and radiology results, and the “Clinical Information System (CIS)”, which contains electronic NICU observation charts and daily progress notes. All case records were reviewed by one neonatal clinician (G.P.G.F) with cross‐checking of coding information with electronic case records to ensure accurate diagnosis. In cases where the diagnosis was in doubt, the original case notes were retrieved for further clarification. Baseline pregnancy and delivery outcomes were documented, including gestational age at birth, mode of delivery, birth weight, newborn sex, Apgar scores at 1 and 5 min, and whether the clinical diagnosis of preeclampsia was established. The primary neonatal outcome was prolonged hospitalisation > 7 days, chosen for its clinical relevance and prevalence in the cohort. Additional neonatal outcomes including small‐for‐gestation, gastrointestinal and metabolic complications—were also examined and hold significant clinical importance in understanding the broader impact of angiogenic imbalance. SGA is defined as birth weight less than the 10th centile (z score < −1.28) for gestation corrected for sex [29], according to gestational age‐specific growth charts for Hong Kong [30]. Respiratory distress syndrome (RDS) is defined by progressive respiratory failure shortly after birth in a preterm or early‐term neonate (manifested by tachypnoea, subcostal retractions and/or grunting and an increase in oxygen requirement), in conjunction with characteristic radiological features of RDS and/or requirement for surfactant therapy [31]. Transient tachypnoea of newborn (TTN) is defined by respiratory distress shortly after delivery with radiographical features of TTN and exclusion of other respiratory pathology. Apnoea of prematurity is defined as the cessation of breathing for 20 s or more, accompanied by bradycardia (heart rate at least 30 beats per minute below resting heart rate) and/or oxygen desaturation (SpO2 < 85%) [32]. Respiratory support is defined as the need for ventilatory support including invasive mechanical ventilation, non‐invasive bilevel positive airway pressure (BIPAP), Continuous Positive Airway Pressure (CPAP), Neurally Adjusted Ventilatory Assist Ventilation (NAVA) or high flow nasal cannula (HFNC). Feeding intolerance is defined as failure to achieve full feeds 14 days after initiating feeding [33]. Clinical sepsis is defined as clinical features suggestive of sepsis (e.g., unstable temperature or fever, poor perfusion) and/or abnormal C‐reactive protein (CRP) or white cell count beyond the normal limits specified for age and laboratory without positive culture results [34, 35]. Hypoglycemia is defined as blood glucose of less than 2.6 mmol/L [36].

### 2.4 Statistical Analysis

Data was analysed using SPSS for Windows version 28.0 (IBM Corp, Armonk, NY, USA). Baseline characteristics such as maternal history and neonatal demographic characteristics were descriptively compared between neonates delivered from pregnancies with an elevated sFlt‐1/PlGF ratio ≥ 85 (high‐risk group) and those from pregnancies with a sFlt‐1/PlGF ratio < 85 (low‐risk group). Quantitative data were summarised and presented as median and interquartile range (IQR) or mean and standard deviation (SD) and 95% Confidence Interval (C.I.) as appropriate, while categorical data were expressed as numbers and percentages. Categorical variables were compared using the Chi‐square test or Fisher's exact test as appropriate. For comparison of continuous data, the Student *t*‐test or Mann Whitney *U* test were used for normally and non‐normally distributed data, respectively.

Logistic regression analysis was performed to evaluate the association between an elevated sFlt‐1/PlGF ratio and various neonatal outcomes with preeclampsia status of the mother and gestational age at delivery as co‐variates. Linear regression analysis was performed to evaluate the association between the sFlt‐1/PlGF ratio and the duration of respiratory and oxygen support, duration of parenteral nutrition (PN), and length of NICU stay, with gestational age at birth and birth weight as co‐variates. The level of significance was set as a two‐sided *p*‐value of less than 0.05.

## 3 Results

**FIGURE 1:**
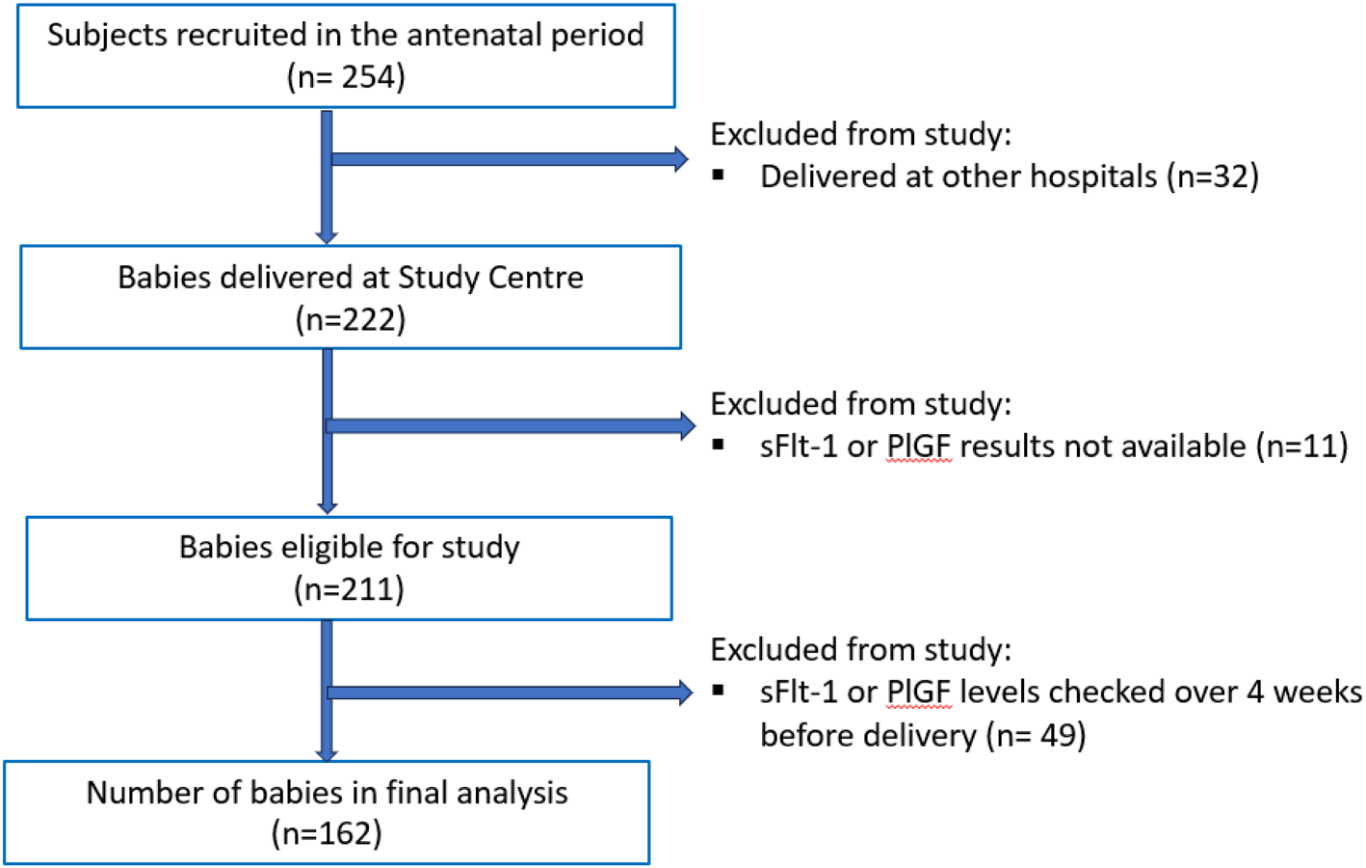
Study recruitment and patient selection flowchart. Flowchart illustrating the recruitment and selection process of study participants. Subjects (pregnant women) were recruited by the Obstetrics team between 20+0 to 36+6 weeks of gestation if they are East Asian ethnicity, aged ≥ 18 years with a singleton pregnancy and with one or more of the following clinical features of suspected preeclampsia (see "Methods"). Among 254 subjects screened for the study, 162 met eligibility criteria and were subsequently included in the final analysis.

A total of 254 mother/baby dyads were included. 92 neonates were excluded with reasons as follows: sFlt‐1 or PlGF levels not available (*n* = 11), sFlt‐1 or PlGF measured more than 4 weeks before delivery (*n* = 49), neonate delivered at another hospital (*n* = 32). Therefore, 162 neonates were included in the final analysis (Figure 1).

There were 56 neonates (34.6%) delivered from pregnancies with an elevated sFlt‐1/PlGF ratio ≥ 85 (high‐risk group) and 106 neonates (65.4%) from pregnancies with a sFlt‐1/PlGF ratio < 85 (low‐risk group). 81 neonates (50%) were born to mothers who developed clinical preeclampsia after recruitment (regardless of biomarker levels). 34 neonates (21%) were delivered before 34 weeks. The baseline maternal and neonatal characteristics of the two groups are shown in Table 1. Maternal characteristics including maternal height, body mass index, ethnicity, percentage of nulliparity, smoking and maternal diabetes mellitus (DM) or gestational DM were similar between the two groups. Compared to the low‐risk group, neonates in the high‐risk group were delivered at a significantly lower median gestational age (34.36 weeks (IQR 31.04–36.00 weeks) vs. 37.00 weeks (IQR 35.57–37.43 weeks) *p* < 0.001), had a lower median birth weight (1846 g (IQR 1156.50 g–2152.50 g) vs. 2487 g (IQR 1977.50 g–2801.25 g), *p* < 0.001) and lower median Apgar scores at 1 min (8.00 (IQR 6.25–9.00) vs. 8.00 (IQR 8.00–9.00), *p* < 0.001) and 5 min (9.00 (IQR 9.00–10.00) vs. 10.0 (IQR 9.00–10.00), *p* = 0.013). Neonates in the high‐risk group also had significantly higher rates of preterm delivery before 37 weeks' gestation (94% vs. 48.1%, *p* < 0.001), maternal clinical preeclampsia (76.7% vs. 35.8%, *p* < 0.001) and Caesarean delivery (80.3% vs. 49.1%, p < 0.001). The median sFlt‐1/PlGF value of the high‐risk group was significantly higher than the low‐risk group [191.18 (IQR 127.84–279.40) vs. 30.59 (IQR 8.96–63.02)]. Sex distribution was similar between the two study groups.

**TABLE 1:** Comparison of baseline characteristics of neonates with elevated sFlt‐1/PlGF and controls.

Baseline demographic features	High‐risk group (sFlt‐1/PlGF ≥ 85, *n* = 56)	Low‐risk group (sFlt‐1/PlGF < 85, *n* = 106)	*p*
**Maternal characteristics**			
Maternal height, cm [Median (IQR)]	158 (155–162)	159 (155–164)	0.09
Maternal BMI [Median (IQR)]	23.53 (21.29–26.75)	25.27 (21.64–27.81)	0.14
Ethnicity Chinese *N* (%)	55 (98.2%)	104 (98.1%)	0.96
Para 0 *N* (%)	38 (67.8%)	61 (57.5%)	0.20
Gestation at sFlt‐1/PlGF analysis (Median (IQR))	33.14 (29.91–35.29)	35.07 (33.29–36.04)	0.01*
Maternal smoking	6 (10.7%)	6 (5.7%)	0.34
Maternal diabetes mellitus (DM)/Gestational DM	12 (21.4%)	12 (11.3%)	0.09
Preeclampsia	43 (76.7%)	38 (35.8%)	< 0.001*
**Neonatal characteristics**			
Gestation (weeks) median (IQR)	34.36 (31.04–36.00)	37.00 (35.57–37.43)	< 0.001*
Prematurity *N* (%)	47 (94.0%)	51 (48.1%)	< 0.001*
Gender male *N* (%)	32 (57.1%)	51 (48.1%)	0.27
Caesarean section *N* (%)	45 (80.3%)	52 (49.1%)	< 0.001*
Birth weight (grams) (median (IQR))	1846 (1156.50–2152.50)	2487 (1977.50–2801.25)	< 0.001*
*Apgar scores (AS)*			
AS at 1 min, median (IQR)	8.0 (6.25–9.00)	8.0 (8.00–9.00)	0.001*
AS at 5 min, median (IQR)	9.0 (9.00–10.00)	10.0 (9.00–10.00)	0.013*
Congenital anomalies *N* (%)	5 (4.7%)^a^	9 (16%)^a^	0.014*

*Note:* The “Low‐risk” group includes all patients with sFlt‐1/PlGF ratios < 85, of whom 78 had ratios < 38, consistent with thresholds validated by the PROGNOSIS and PROGNOSIS Asia studies to rule out preeclampsia. * denotes significant *p*‐value.

Abbreviations: PlGF, placental growth factor; sFlt‐1, fms‐like tyrosine kinase 1.

^a^ Congenital anomalies: Dysmorphic features (2), Cardiac anomalies (2), Gastrointestinal anomalies (2), Renal/genitourinary anomalies (3), Skeletal anomalies (3), Metabolic anomaly (1), Congenital deafness (1).

Neonatal outcomes stratified by angiogenic risk group, including event counts, absolute risks, risk differences, and adjusted odds ratios are shown in Table 2. Logistic regression analysis showed that after adjustment for gestational age at birth, clinical preeclampsia, and newborn sex, neonates in the high‐risk group had significantly higher incidence of prolonged hospitalisation > 7 days (aOR 3.62, 95% CI 1.20–10.87, *p* = 0.02), with an absolute risk difference of +47.2% (78.6% vs. 31.4%). Other adverse neonatal outcomes with significantly increased risks in the high‐risk group included small for gestational age (SGA) (aOR 2.52, 95% CI 1.12–5.60, *p* = 0.03), feeding intolerance (aOR 3.03, 95% CI 1.01–9.09, *p* = 0.04), need for parenteral nutrition (PN) (aOR 11.19, 95% CI 2.23–56.29, *p* = 0.003), prolonged PN > 7 days (aOR 6.92, 95% CI 1.43–33.39, *p* = 0.02), and hospital stay > 30 days (aOR 7.04, 95% CI 1.08–45.80, *p* = 0.02). Absolute risk differences for these outcomes ranged from +25.9% to +46.1%. Adjusted marginal probabilities closely mirrored actual event rates, suggesting minimal confounding by covariates.

**TABLE 2:** Adjusted odds ratios and absolute risk estimates for neonatal outcomes by sFlt‐1/PlGF ratio group.

Neonatal outcome	High‐risk group (sFlt‐1/PlGF ≥ 85, *n* = 56)	Low‐risk group (sFlt‐1/PlGF < 85, *n* = 106)	Absolute risk difference (%)^a^	Adjusted odds ratio (95% CI)^b^	*p*
Small for gestation (SGA)	31/56 (55.4%)	29/106 (27.4%)	+28.0%	2.52 (1.12–5.60)	0.03*
Respiratory distress syndrome (RDS)	22/56 (39.3%)	11/106 (10.4%)	+28.9%	1.01 (0.22–4.54)	0.99
Transient tachypnoea of newborn (TTN)	2/56 (3.6%)	4/106 (3.8%)	−0.2%	0.65 (0.09–4.74)	0.68
Apnoea of prematurity	20/56 (35.7%)	8/106 (7.5%)	+28.2%	1.54 (0.48–4.76)	0.47
Respiratory support (CPAP/BIPAP)	25/56 (44.6%)	16/106 (15.1%)	+29.5%	2.13 (0.72–6.25)	0.17
Feeding intolerance	31/56 (55.4%)	14/106 (13.2%)	+42.2%	3.03 (1.01–9.09)	0.04*
Need for parenteral nutrition (PN)	30/56 (53.6%)	8/106 (7.5%)	+46.1%	11.19 (2.23–56.29)	0.003*
Prolonged PN (> 7 days)	27/56 (48.2%)	8/106 (7.5%)	+40.7%	6.92 (1.43–33.39)	0.02*
Hypoglycaemia	29/56 (51.8%)	38/106 (35.8%)	+16.0%	0.69 (0.32–1.51)	0.36
Clinical sepsis	18/56 (32.1%)	8/106 (7.5%)	+24.6%	1.41 (0.41–5.00)	0.58
Admission to NICU	30/56 (53.6%)	18/106 (17.0%)	+36.6%	1.79 (0.69–4.60)	0.20
Hospital stay > 7 days	44/56 (78.6%)	33/105 (31.4%)	+47.2%	3.62 (1.20–10.87)	0.02*
Hospital stay > 30 days	22/56 (39.3%)	6/106 (5.7%)	+33.6%	7.04 (1.08–45.80)	0.02*

*Note:* Neonatal outcomes are stratified by angiogenic risk group based on sFlt‐1/PlGF ratio (≥ 85 vs. < 85). Event counts and percentages are shown for each group.

^a^ Absolute risk differences represent the difference in event rates between high‐risk and low‐risk groups.

^b^ Adjusted odds ratios (aORs) and 95% confidence intervals (CIs) are derived from logistic regression models controlling for gestational age at birth, clinical preeclampsia, and newborn sex.

* Statistical significance at *p* < 0.05.

Gestational age at birth also showed a significant association with all adverse neonatal outcomes but there was no significant association between clinical preeclampsia and various neonatal outcomes after adjustment for confounders.

Linear regression analysis using gestational age at birth, the sFlt‐1/PlGF ratio and birth weight as continuous variables (Table 3) showed that an increase in the sFlt‐1/PlGF ratio was associated with significantly longer durations of respiratory support (standardised β = 0.22, *p* = 0.01) and oxygen supplement (standardised β = 0.21, *p* = 0.008); a longer duration on PN (standardised β = 0.01, *p* = 0.01); longer length of NICU stay (standardised β = 0.33, *p* < 0.001) and longer total hospital stay (standardised β = 0.24, *p* = 0.01). Each unit increase in the sFlt‐1/PlGF ratio corresponded to an increase in days on ventilator support (0.054), oxygen (0.064), PN (0.021), NICU stay (0.096), and total hospital stay (0.079).

**TABLE 3:** Linear regression analysis comparing sFlt‐1/PlGF, gestational age at birth, birth weight and neonatal outcomes.

Outcome	sFlt‐1/PlGF ratio			Gestational age at birth			Birth weight		
	B‐coefficient (95% C.I.)	Standardised β	*p*	B‐coefficient (95% C.I.)	Standardised β	*p*	B‐coefficient (95% C.I.)	Standardised β	*p*
Number of days on respiratory support	0.054 (0.013–0.095)	0.220	0.01*	−8.472 (−12.058 to −4.885)	−0.586	< 0.001*	0.008 (−0.006 to 0.023)	0.144	0.26
Number of days on oxygen	0.064 (0.017–0.112)	0.210	0.008*	−8.718 (−12.871 to −4.656)	−0.535	< 0.001*	0.009 (−0.008 to 0.026)	0.135	0.31
Number of days on PN	0.021 (0.005–0.036)	0.008	0.01*	−4.719 (−6.079 to −3.359)	−0.688	< 0.001*	0.001 (−0.005 to 0.006)	0.020	0.85
Length of stay in NICU (days)	0.096 (0.052–0.140)	0.326	< 0.001*	−5.833 (−9.684 to −1.983)	−0.373	0.003*	0.001 (−0.015 to 0.016)	0.011	0.93
Total length of stay (days)	0.079 (0.036–0.121)	0.242	0.01*	−8.696 (−12.394 to 4.999)	−0.506	< 0.001*	−0.004 (−0.019 to 0.011)	−0.062	0.85

*Note:* Linear regression analysis showing the association of sFlt‐1/PlGF, gestation and preeclampsia with various neonatal outcomes. sFlt‐1/PlGF and gestation are entered as continuous variables. Multicollinearity analysis indicated no significant collinearity between co‐variates. * denotes significant *p*‐value.

Abbreviations: NICU, neonatal intensive care unit; PlGF, placental growth factor; PN, parenteral nutrition; sFlt‐1, fms‐like tyrosine kinase 1.

## 4 Discussion

### 4.1 Main Findings

Neonates delivered from mothers with suspected preeclampsia and an elevated sFlt‐1/PlGF ratio ≥ 85 during pregnancy have significantly increased risks of adverse neonatal outcomes, namely prolonged hospitalisation > 7 days, SGA, feeding intolerance, requirements for PN, increased duration of oxygen and ventilatory support; prolonged NICU and hospital stay. These associations were statistically significant and consistent across multiple regression models.

### 4.2 Interpretations

Preeclampsia was traditionally diagnosed according to maternal symptoms of hypertension, proteinuria, and end‐organ dysfunction. Recently, advances in research have revealed more complex pathophysiology involving spiral artery re‐modelling and placental dysfunction, angiogenic imbalance [28, 37], inflammation, and fetal epigenetic changes [38]. Placental biomarkers sFlt‐1 and PlGF have emerged as reliable indicators of disease risk, and the sFlt‐1/PlGF ratio is integrated into clinical obstetrics care [39]. Biomarker‐based approaches offer improved risk assessment [40] and may enhance neonatal care by enabling earlier identification of high‐risk infants and guiding management.

Clinical preeclampsia was not independently linked to neonatal outcomes in this study, likely due to its limited sensitivity in detecting placental dysfunction. Mild cases may not fulfill the diagnostic criteria of preeclampsia but still affect fetal health. In contrast, elevated sFlt‐1/PlGF ratios offer a more precise reflection of placental pathology and are a potentially superior marker of fetal risk. An elevated sFlt‐1/PlGF ratio reflects ongoing placental dysfunction and compromised nutrient and oxygen supply to the fetus, causing adverse outcomes in the neonate. From a pathophysiological perspective, the sFlt‐1/PlGF ratio should be closely associated with neonatal outcomes [28, 37].

This study shows that an elevated sFlt‐1/PlGF ratio is significantly associated with adverse neonatal gastrointestinal outcomes, including feeding intolerance and prolonged PN use. These complications may be due to abnormalities in placental blood flow, causing decreased splanchnic and gastrointestinal perfusion. Pelica et al. have demonstrated that preterm neonates exposed to preeclampsia had decreased peak systolic and end‐diastolic velocity in the superior mesenteric artery compared to preterm controls [41, 42], indicating compromised gastrointestinal perfusion in preeclampsia, independent of prematurity. For these neonates, neonatologists should consider avoiding over‐aggressive feeding advancement, using breast milk and avoiding artificial formulas whenever possible. Further research utilising neonatal ultrasound Doppler studies on splanchnic blood flow may be able to provide useful information regarding ongoing gastrointestinal perfusion status [41, 42].

Linear regression analyses have helped to quantify the effect size of changes in the sFlt‐1/PlGF ratio on various aspects of neonatal outcomes in this study. Each unit increase in the sFlt‐1/PlGF ratio corresponds to an increase in the number of days on ventilator support, oxygen, PN, length of NICU stay and total length of stay of 0.054, 0.064, 0.021, 0.096 0.079 days respectively. Although gestational age at birth was also strongly associated with adverse neonatal outcomes, the interaction between gestation and biomarkers is complex and adjusted associations should be interpreted cautiously. Angiogenic biomarker results can influence obstetric decisions; for example, obstetricians may opt for earlier/preterm delivery in cases with very high sFlt‐1/PlGF ratios. Further research with larger cohorts and mediation analysis is needed to clarify this relationship.

Our study shows that SGA is significantly associated with an elevated sFlt‐1/PlGF ratio. Numerous studies have shown a high incidence of postnatal growth failure in SGA neonates [43, 44]. Meticulous nutritional calculations, as well as growth and body composition monitoring in these neonates are crucial, as suboptimal catch‐up growth would have adverse effects on neurodevelopment [45], whilst excessive growth is associated with cardiovascular and metabolic disorders in later life [46]. A multidisciplinary approach involving neonatologists and nutritionists is recommended to optimise nutrition.

On the other hand, respiratory complications including RDS, TTN and apnoea of prematurity are significantly associated with prematurity but not with elevated sFlt‐1/PlGF ratio or preeclampsia. This is because RDS is caused by surfactant deficiency [31], whilst TTN is due to delayed clearance of pulmonary fluid [47] and apnoea of prematurity is caused by immature central respiratory drive [48], all of which are directly related to prematurity. These results indicate that prematurity plays a more significant role than placental dysfunction in the pathogenesis of certain neonatal respiratory complications.

A combination of prematurity, SGA and poor feeding tolerance will all contribute to an increased duration of hospital stay for the neonate with increased demands on health‐care resources and added stress for families. A good understanding of the potential neonatal problems and proactive management can facilitate safe and timely discharge.

### 4.3 Implications for Clinical Practice

With close communication between the obstetrics and neonatal teams, perinatal and antenatal information has been increasingly incorporated into neonatal management. For example, risk factors for perinatal sepsis are incorporated into “Neonatal sepsis algorithms” [49, 50] to guide sepsis screening. The sFlt‐1/PlGF ratio can be considered for incorporation into neonatal practice to guide neonatal management. Future research could involve the development of AI algorithms to integrate information such as the sFlt‐1/PlGF ratio, antenatal ultrasound parameters, placental pathology and neonatal characteristics to predict adverse neonatal outcomes. Although epidemiological studies have supported the Developmental Origins of Health and Disease (DoHAD) theory of fetal origins of adult disease in adult offspring of preeclamptic pregnancies [51], the timing, pathophysiology and aggravating factors for adult disease are not known. Prospective large‐scale studies will generate evidence‐based information on the mechanisms affecting long‐term health and enable appropriate and timely primary prevention.

### 4.4 Strengths and Limitations

As far as we know, this is the largest and most comprehensive study on angiogenic markers and neonatal outcomes. The strength of this study is that all pregnant mothers were recruited prospectively, and analysis of angiogenic markers was performed in accordance with a standardised protocol. All NICU data, laboratory results and clinical notes were entered prospectively and stored in our electronic CIS system with printed copies filed in the case notes. Diagnosis and procedure coding were entered prospectively and reviewed by the neonatal team during hospitalisation and on discharge. All case records were reviewed by one neonatal clinician (G.P.G.F.) using both electronic and original case notes to ensure accuracy. Multivariate logistic regression and linear regression analysis were performed in the statistical analysis to control for confounders and to ensure robust results.

There are several limitations in this study. As this is a retrospective study, some neonatal parameters, including cardiac function, haemodynamics and cerebral blood flow, cannot be delineated. Aye et al. demonstrated changes in ventricular mass in neonates from preeclamptic pregnancies [52], whilst abnormal blood pressure and heart rate were found in other studies [53]. Serial measurements of sFlt‐1/PlGF before delivery to observe trends are also not available. Prospective studies investigating these parameters are needed. Neonatal data were only available up to hospital discharge, and long‐term outcomes of these neonates are not known. Finally, it is a single‐centre study with a relatively small sample size. The findings in this study would benefit from confirmation in larger multicentre cohorts. Long‐term prospective studies are needed to guide clinical implementation and direct future research.

## 5 Conclusion

This study demonstrates that an elevated sFlt‐1/PlGF ratio in pregnancies complicated by suspected preeclampsia is significantly associated with a spectrum of adverse neonatal outcomes, including small‐for‐gestational‐age, gastrointestinal and metabolic complications, and prolonged hospitalisation. The sFlt‐1/PlGF ratio can be utilised as a relevant biomarker not only for maternal risk stratification but also for anticipating neonatal morbidity and has the potential to be integrated into neonatal care planning. Large‐scale prospective studies are needed to further delineate the neonatal effects of angiogenic imbalance, and to explore how incorporation of angiogenic biomarkers information can enhance neonatal management, optimise resource allocation, and improve neonatal outcomes.

## Author Contributions

G.P.G.F., L.N.‐H., D.J.S. and L.C.P. designed the research study; G.P.G.F., L.N.‐H., I.Y.M.W., C.S.L.L. and D.J.S. collected and prepared the data; G.P.G.F., K.Y.Y.C. and L.C.P. analysed the data; G.P.G.F., L.N.‐H. and L.C.P. drafted the original manuscript, H.S.L. provided expert advice; G.P.G.F., L.N.‐H., K.Y.Y.C. and L.C.P. revised the manuscript; L.C.P. supervised the study. All authors have read and approved the final version of the manuscript.

## Funding

The study was provided by the Health and Medical Research Fund (HMRF Project No. 08191526) and the Ministry of Science and Technology (MOST), China (No. 2021YFC2701600).

## Conflicts of Interest

L.C. Poon has received speaker fees and consultancy payments from Roche Diagnostics and Ferring Pharmaceuticals. In addition, she has received in‐kind contributions from Roche Diagnostics, Revvity Inc. (formerly PerkinElmer Life and Analytical Sciences), Thermo Fisher Scientific, Ningbo Aucheer Biological Technology Co. Ltd., and GE Healthcare. D.S. Sahota has received in‐kind contributions from Revvity Inc. (formerly PerkinElmer Life and Analytical Sciences), Thermo Fisher Scientific, Roche Diagnostics, Diabetomics and Ningbo Aucheer Biological Technology Co. Ltd. Other authors report no conflicts of interest.

## Data Availability

The data that support the findings of this study are available from the corresponding author upon reasonable request.
